# Leverage of an Existing Cervical Cancer Prevention Service Platform to Initiate Breast Cancer Control Services in Zambia: Experiences and Early Outcomes

**DOI:** 10.1200/JGO.17.00026

**Published:** 2017-09-08

**Authors:** Leeya F. Pinder, Ronda Henry-Tillman, David Linyama, Victor Kusweje, Jean-Baptiste Nzayisenga, Aaron Shibemba, Vikrant Sahasrabuddhe, Kennedy Lishimpi, Mulindi Mwanahamuntu, Michael Hicks, Groesbeck P. Parham

**Affiliations:** **Leeva F. Pinder**, **David Linyama**, **Jean-Baptiste Nzayisenga**, **Aaron Shibemba**, **Mulindi** **Mwanahamuntu**, and **Groesbeck P. Parham**, University of Zambia School of Medicine; **Kennedy Lishimpi**, Cancer Diseases Hospital, Lusaka; **Victor Kusweje**, Kabwe General Hospital, Kabwe, Zambia; **Leeya F. Pinder**, **Michael Hicks**, and **Groesbeck P. Parham**, University of North Carolina at Chapel Hill, Chapel Hill, NC; **Ronda Henry-Tillman**, University of Arkansas for Medical Sciences, Little Rock, AR; and **Vikrant Sahasrabuddhe**, National Cancer Institute, Bethesda, MD.

## Abstract

**Purpose:**

In 2005, the Cervical Cancer Prevention Program in Zambia (CCPPZ) was implemented and has since provided cervical cancer screen-and-treat services to more than 500,000 women. By leveraging the successes and experiences of the CCPPZ, we intended to build capacity for the early detection and surgical treatment of breast cancer.

**Methods:**

Our initiative sought to build capacity for breast cancer care through the (1) formation of a breast cancer advocacy alliance to raise awareness, (2) creation of resource-appropriate breast cancer care training curricula for mid- and high-level providers, and (3) implementation of early detection and treatment capacity within two major health care facilities.

**Results:**

Six months after the completion of the initiative, the following outcomes were documented: Breast health education and clinical breast examination (CBE) services were successfully integrated into the service platforms of four CCPPZ clinics. Two new breast diagnostic centers were opened, which provided access to breast ultrasound, ultrasound-guided core needle biopsy, and needle aspiration. Breast health education and CBE were provided to 1,955 clients, 167 of whom were evaluated at the two diagnostic centers; 55 of those evaluated underwent core-needle biopsy, of which 17 were diagnosed with invasive cancer. Newly trained surgeons performed six sentinel lymph node mappings, eight sentinel lymph node dissections, and 10 breast conservation surgeries (lumpectomies).

**Conclusion:**

This initiative successfully established clinical services in Zambia that are critical for the early detection and surgical management of breast cancer.

## INTRODUCTION

Breast cancer in sub-Saharan Africa is characterized by low but rapidly increasing disease incidence,^1,2^ late-stage presentation, high mortality, and a heavy burden among younger women.^3,4^ Increasing longevity and the replacement of traditional African lifestyles with Western patterns of food consumption, childbearing, and breastfeeding undergird the increase in disease incidence.^2^ Much of the late-stage presentation and high mortality rates can be attributed to system-level barriers to early detection and treatment.^5^ Other contributing factors include lack of awareness of early signs and symptoms of the disease^6,7^; a general belief that cancer has a supernatural origin and is always fatal^8^; use of traditional therapies before or in lieu of seeking modern treatment^9-11^; and fear of spousal abandonment after mastectomy.^12^ The need for a high-impact, scalable, breast cancer control model that has the inherent capability to adjust to the myriad of complex situations and circumstances that women in low-resource environments have to negotiate is paramount. In this paper, we describe experiences and initial results of a public-private demonstration project to expand access to breast cancer control services in Zambia. It was built on the framework of a decade-long program that successfully integrated cervical cancer prevention interventions within service platforms used to deliver HIV/AIDS care and treatment in Zambia.

## METHODS

Since 2005, the Zambian government, with support from the US President’s Emergency Plan for AIDS Relief, which is funded through the US Centers for Disease Control Prevention, and in partnership with local and US university-based public health oncology experts and advocates, has facilitated the implementation of a public-sector cervical cancer prevention intervention—Cervical Cancer Prevention Program in Zambia (CCPPZ). Initially focused on the provision of cervical cancer screening and treatment services to HIV-infected women, it soon expanded services to all women regardless of HIV status as a result of patient demand. During the past decade, albeit with resources disproportionate to demand, cervical cancer prevention services have been scaled nationally to 60 government-operated public health clinics, at which more than 500,000 women have been screened since program inception and 50,000 are screened annually. An analysis of the first 100,000 women enrolled in the program revealed a screen-to-detection ratio of 56 to 1 for high-grade cervical precancer, an overall treatment rate of 70% for women diagnosed with precancerous lesions, and a shift in the percentage of early-stage invasive cervical cancers from 20% to 42%.^13^ As of February 2015, CCPPZ was officially integrated into the government’s public health system with an official desk—National Coordinator of Cancer Prevention—now located at the Zambian Ministry Health.

Our major objectives were to leverage the successes and experiences of Zambia’s cervical cancer prevention program and to use direct participation at the point of care to build capacity for detection and surgical treatment of breast cancer in Zambia. We also assessed the clinical advancements that resulted from the capacity-building activities. The following cluster of activities were conducted to implement the initiative:

1. Creation of an alliance of advocacy groups: With support from the Susan G. Komen Foundation, the first step was to facilitate the formation of an alliance of the five existing breast and cervical cancer advocacy groups in Zambia into a consortium—CAPRAZ (Cancer Prevention Alliance Zambia)—with the following goals (1) intensify breast and cervical cancer awareness campaigns in the community to dispel myths and misconceptions surrounding the disease and its treatment; (2) ensure that breast and cervical cancer health promotion messages were evidence based and contextually relevant, and (3) coordinate and provide technical support for awareness activities of member groups.
2. Assess capacity: After the organization of CAPRAZ into a functional nonprofit organization, the next step was to assess breast cancer early detection and diagnostic and surgical treatment capacities. Toward this end, breast oncology consultants from two university medical centers in the United States (University of Arkansas and University of North Carolina, Chapel Hill) were invited to Zambia to assess breast cancer care capacity at the University Teaching Hospital in Lusaka Province (the country’s single tertiary medical facility that serves as its primary center for postgraduate medical training) and Kabwe General Hospital in Central Province (a large, rural provincial medical center representative of the types of health facilities that have been prioritized by the Zambian National Cancer Control Strategic Plan as high-priority sites for the expansion of cancer control services). Upon receipt of approval from relevant government agencies, the consultants visited the two designated sites and worked daily alongside local health professionals in their breast clinics, surgical theaters, and postoperative wards. They also attended hospital-based educational conferences, convened meetings with key academic stakeholders and professional health care providers, gave formal lectures on breast cancer diagnosis and management to faculty and staff, and hosted informal social gatherings. From the assessment, it was determined that the largest and most critical deficits in the breast cancer care pathway at the two facilities were lack of capacity to perform the following: (1) clinical breast examination (CBEs), (2) breast ultrasound, (3) ultrasound-guided core needle biopsy of palpable breast masses, and (4) conservative surgical treatment of breast cancer. Other weaknesses and strengths are listed in Table 1.
3. Curriculum development: The team of US consultants, in partnership with Zambian health care professionals, used information from the assessment and knowledge of the local context to develop a training curriculum aimed at building capacity in these four areas. Once developed, the curriculum was vetted by senior members of the Zambian Ministry of Health’s Technical Working Group for purposes of modification and approval.
4. Implementation of the curriculum: Four months after their initial visit, the US oncology consultants returned to Zambia and implemented the training curriculum (Table 2), in the form of a 1-week long practicum at the University Teaching Hospital, entitled “A Model for Expansion of Breast Cancer Care in Zambia.” Training consisted of a combination of didactics and hands-on mentoring and evaluation by US in the breast clinics and surgical theaters at the University Teaching Hospital and Kabwe General Hospital.
5. Development of benchmarks for evaluation: The following benchmarks were established for impact evaluation of the initiative:
  (1) Cervical cancer screening nurses working in government-operated primary health centers could adequately deliver breast health awareness education and CBE.
  (2) Local radiographers (technicians) could accurately perform ultrasonographic evaluation of palpable breast masses.
  (3) General surgeons could safely and effectively perform ultrasound-guided core needle biopsy and needle aspiration of breast masses, axillary sentinel lymph node (SLN) mapping with blue dye, SLN dissection, and breast lumpectomy.
  (4) Breast diagnostic centers would be established at each of the two facilities described as assessment sites, with the capacity to perform CBE, breast ultrasound, and ultrasound-guided core needle biopsy of palpable breast masses.
  (5) Data collection platforms would be established in each clinic.
  (6) Referral pathways would be developed between the breast diagnostic clinics and either the departments of general surgery at the respective hospitals or the national cancer center (Cancer Diseases Hospital) in Lusaka; referral pathways also would be developed for chemotherapy and radiation at the national cancer center in Lusaka.
  (7) Educational activities implemented by CAPRAZ in the catchment areas surrounding the two hospitals would increase awareness of the signs and symptoms of breast cancer and encourage women with breast concerns to seek care from local health facilities. Member groups also would begin to increase their participation in local breast cancer initiatives, such as fundraisers, educational seminars, public lectures, and awareness campaigns.

**Table 1:**
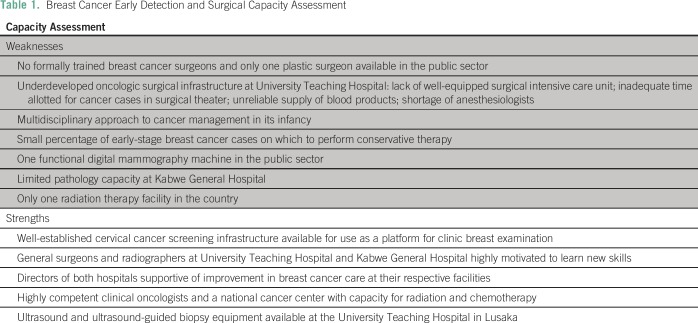
Breast Cancer Early Detection and Surgical Capacity Assessment

**Table 2:**
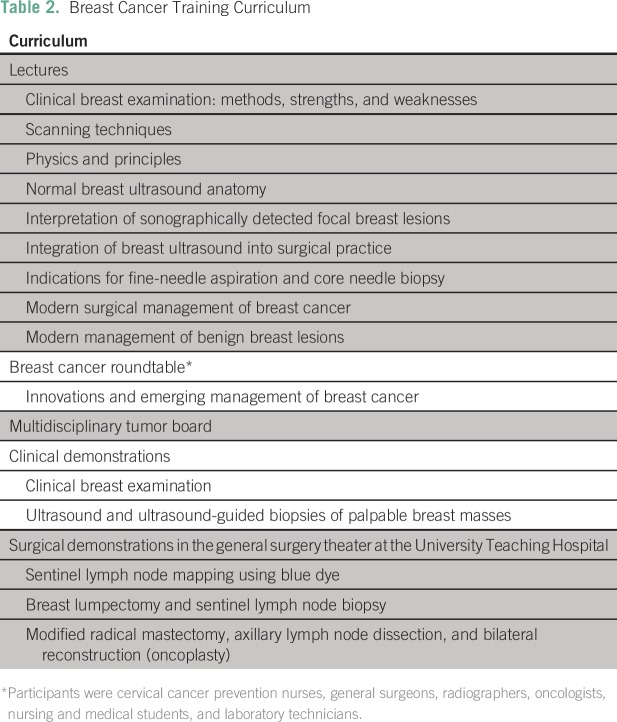
Breast Cancer Training Curriculum

## RESULTS

**Fig 1:**
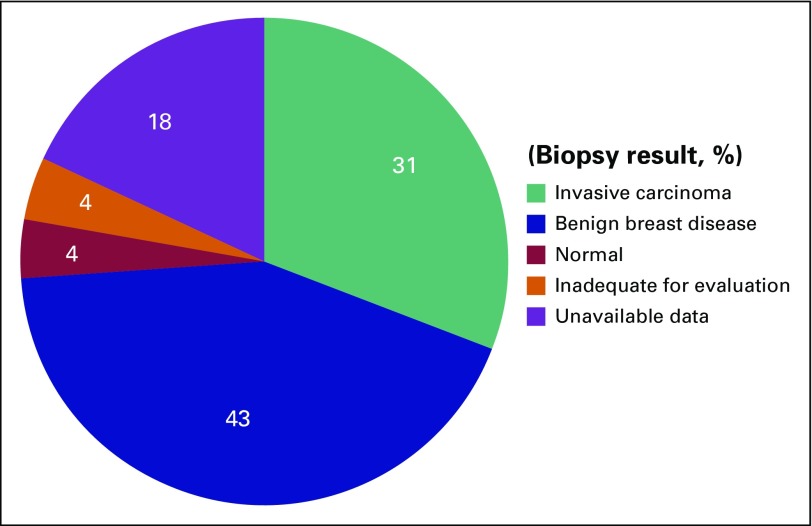
Breast biopsy results (N = 55).

A 6-month evaluation of local activities by US consultants revealed the following:

1. Four cervical cancer screening clinics at the primary health care level—one in Kabwe and three in Lusaka provinces—had successfully integrated breast cancer education and CBE into their routine service platforms.
2. Two new breast cancer diagnostic centers were operational—one at Kabwe General Hospital and the other at University Teaching Hospital, and each provided CBE, breast ultrasound, ultrasound-guided core needle biopsy, and needle aspiration.
3. A total of 1,955 women had undergone CBE in the four primary health centers, of which 256 (13.1%) had palpable breast masses (Table 3). All were referred to one of the two new breast diagnostic centers for additional evaluation; of these, 167 (65.2%) actually attended. A secondary CBE at the referral centers confirmed palpable breast masses in 59 (35.3%) of those who underwent the examination (Table 3).
4. Ultrasound-guided biopsies were indicated and performed in 55 of the 59 women who had confirmed palpable breast masses (Table 3). Pathology results were available for 45 (81.8%) of the 55 who underwent biopsy (Fig 1). Breast cancer was confirmed in 17 (37.8%), and benign breast lesions in 24 (43.6%). The remaining four pathology results were classified as either normal or inadequate for diagnosis. The median (± standard deviation) age of women diagnosed with breast cancer was 40 years (± 13.4 years). The median diameter of breast cancer masses detected by ultrasound was 2.9 cm. Fibroadenoma (n = 10) was the most commonly reported benign lesion (Table 4).
5. Six SLN mappings, eight SLN dissections, and 20 breast conservation surgeries (lumpectomies) were performed independently by the Zambian general surgeons. The histologic margin status of lumpectomy surgical specimens was not reported. No surgical complications were reported (Table 5).

**Table 3:**
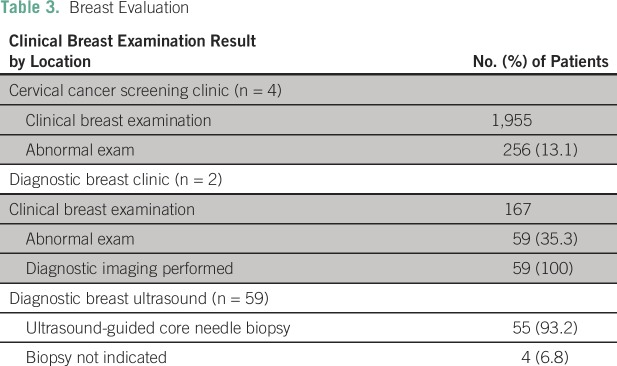
Breast Evaluation

**Table 4:**
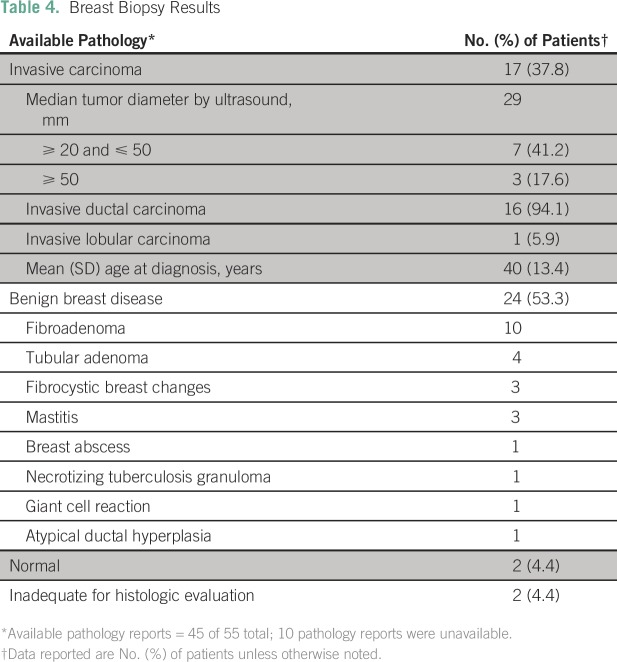
Breast Biopsy Results

**Table 5:**
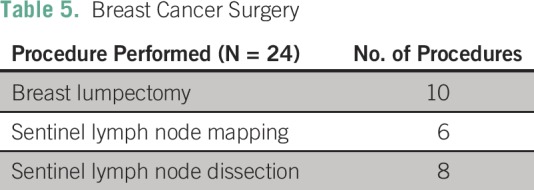
Breast Cancer Surgery

## DISCUSSION

Breast cancer is projected to increase in low- and middle-income countries (LMICs) during the next two decades. The vast majority of women who live in these settings will die from their disease primarily because of the advanced stage at which they are diagnosed and lack of access to high-quality treatment services. In addition, attempts to develop effective breast cancer care service platforms in such settings are made difficult by widespread myths and misconceptions about the disease as well as by competing health care priorities. We took advantage of prior investments in the integration of cervical cancer screening within HIV services to launch an approach to capacity building for the early detection and surgical treatment of breast cancer in Zambia.

Cervical cancer screening nurses were trained to provide breast cancer education and perform CBE on appropriately aged asymptomatic and symptomatic women who attended their clinics for cervical cancer prevention services. Although the most effective means for screening is mammography,^14^ population-based mammographic screening requires resources generally not widely available in LMICs. In addition, its benefit-to-harm ratio has been questioned recently, particularly in women younger than 50 years old—the age group that produces the majority of breast cancers in LMICs.^3,15^ Although there is controversy about the benefits of CBE,^16-19^ there is developing evidence that it has the potential to downstage disease. In an ongoing cluster-randomized trial in India, CBE, when performed by community health care workers, detected more (18.8 *v* 8.1 per 100,000 women) early-stage cancers (stage I to IIA) in intervention versus control villages.^20^

Two new diagnostic breast centers were established and staffed with radiographers and general surgeons who were trained in ultrasound and ultrasound-guided biopsy, respectively. Ultrasound is recommended as an appropriate tool for the evaluation of women with palpable breast masses in LMICs, given its relatively low cost compared with other imaging technologies, value as an adjunct to CBE, and ability to support minimally invasive diagnostic procedures.^21,22^ Ultrasound, however, is dependent on the quality and reliability of the equipment and the experience of operators for accuracy in the diagnosis of breast lesions.^23^ When used effectively, it can distinguish simple cysts from solid masses, provide an estimation of the likelihood of malignancy, and be used to aid in biopsy for pathologic diagnosis.^24^

Ultrasound-guided core needle biopsies are performed to determine the etiology of palpable breast masses and are indicated on the basis of the sonographic characteristics of the mass, such as shape, orientation, margin, echo pattern, posterior acoustics, and boundary.^25^ Compared with open incisional and excisional biopsy, core-needle biopsy is less invasive, less expensive, causes minimal scarring, and can be performed on an outpatient basis.^26^ Core-needle biopsy also has the benefit of provision of adequate tissue for determination of histologic grade; vascular and lymphatic invasion; estrogen and progesterone receptor statuses; and the presence of molecular tumor markers, such as *HER2*/*neu*.^27^ These prognostic variables may help triage surgical interventions, such as breast-conservation therapy,^28^ use of SLN biopsy, or axillary lymph node dissection.^27^

Surgery is the cornerstone of breast cancer care and is the most widely available therapy for breast cancer in LMICs. Modified radical mastectomy (MRM) is the most commonly performed surgical procedure for breast cancer in these settings, largely as a result of late-stage presentation of disease. Although general surgeons in Zambia were well versed in performance of MRMs, their exposure to breast conservation surgery (lumpectomy) and axillary SLN mapping and dissection techniques was limited. The number of surgical theaters was inadequate, and not enough time was allocated to accommodate all of the surgical cases that had been scheduled for surgical demonstrations by visiting oncologists, which resulted in a less-than-desired number for hands-on training. This ultimately limited the training the surgeons received in conservative surgery techniques. Because such a large percentage of women presented with advanced-stage disease, limited numbers of early-stage breast cancer cases were available on which to demonstrate breast lumpectomy and SLN mapping/dissection. Despite these challenges, the surgeons were able to independently perform breast conservation surgery and SLN mapping and dissection techniques. Breast conservation surgery for early-stage disease is safe and has the same overall survival as mastectomy.^29^ It has the added benefit of better cosmetic appearance, which is important, given the impact of cultural beliefs and stigma on treatment-seeking behavior.^12,30^ MRMs, however, will remain the mainstay of treatment of breast cancer in LMICs for the foreseeable future given the limitations imposed by the lack of radiotherapy services and inefficient resources for effective follow-up programs.^31^ Complete level-1 and -2 axillary dissections typically are performed during breast cancer surgeries in LMICs because of advanced-stage disease or the inability to perform less invasive axillary staging in early-stage disease. With proper training, however, SLN mapping and sampling can be performed, which may increase the accuracy of axillary staging in breast cancer and reduce morbidity associated with complete axillary dissections.^32^ This will become a necessary skill as more cancers are diagnosed at an early stage.

We used direct participation at the point of care to inform the construction and implementation of an approach to capacity building for the early detection and surgical treatment of breast cancer in Zambia (Table 6). As an overall impact, CBEs and breast education are now performed by cervical cancer screening nurses at select cervical cancer prevention clinics in Zambia. Two new diagnostic breast centers have been established that provide modern diagnostic services in the form of breast ultrasound and ultrasound-guided biopsies. Efforts are underway to make them sustainable by using resources provided through local public-private partnerships. General surgeons at two of the country’s largest health facilities are now aware of how to perform breast-conserving surgery, including lumpectomy and axillary SLN mapping/dissection for early-stage disease. Two general surgeons are undergoing 6 months of additional hands-on training under the tutelage of breast cancer surgeons in South Africa, through a south-south educational collaboration sponsored by the Susan G. Komen Foundation and the Zambian Ministry of Health. A third has been accepted into a formal surgical oncology fellowship in India. Active participation in local clinics and surgical theaters by the US oncology experts provided them with unique insights into the challenges of breast cancer control in Zambia and will serve to shape future surgical training collaborative efforts.

**Table 6:**
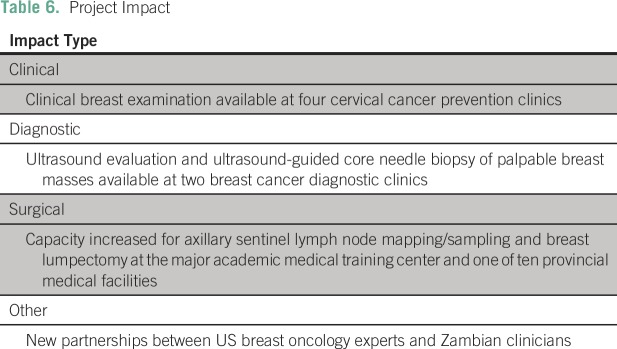
Project Impact

Future initiatives will concentrate on the expansion of breast cancer early detection and surgical treatment services throughout the country. This will require increased training of nurses, radiographers, and surgeons as well as an effective system to monitor quality, access timely and accurate pathology services, and obtain patient follow-up information. As awareness is increased and CBE is more widely implemented, early-stage presentation will become more prevalent and the demand for breast conservation surgical skills, as well as those for breast reconstruction, will increase.

In conclusion, system-level barriers to early detection and treatment of cancer continue to plague LMICs. We leveraged an existing cervical cancer prevention platform to introduce breast cancer education, detection, and surgical treatment services by investing in the development of local clinical expertise. Overall, this initiative has increased breast care capacity in Zambia and, if properly scaled, can serve to improve the diagnosis and management of early-stage breast cancer at a national level, which thereby reducing breast cancer morbidity and mortality.

## References

[1] Parkin DM Nambooze S Wabwire-Mangen F et al: Changing cancer incidence in Kampala, Uganda, 1991-2006. Int J Cancer 126:1187-1195, 2010 19688826 10.1002/ijc.24838

[2] Sylla BS Wild CP: A million Africans a year dying from cancer by 2030: What can cancer research and control offer to the continent? Int J Cancer 130:245-250, 2012 21796634 10.1002/ijc.26333 PMC3244688

[3] Ferlay J Soerjomataram I Dikshit R et al: Cancer incidence and mortality worldwide: Sources, methods and major patterns in GLOBOCAN 2012. Int J Cancer 136:E359-E386, 2015 25220842 10.1002/ijc.29210

[4] Sankaranarayanan R Swaminathan R Brenner H et al: Cancer survival in Africa, Asia, and Central America: A population-based study. Lancet Oncol 11:165-173, 2010 20005175 10.1016/S1470-2045(09)70335-3

[5] Kingham T.P. Alatise OI Vanderpuye V et al: Treatment of cancer in sub-Saharan Africa. Lancet Oncol 14:e158-e167, 2013 23561747 10.1016/S1470-2045(12)70472-2

[6] Machlin A, Wakefield M, Spittal M, et al: UICC Special Report: Cancer-related beliefs and behaviours in eight geographic regions. http://old.uicc.org/templates/uicc/pdf/special%20reports/survey%20report.pdf

[7] Maree J Wright S Lu X: Breast cancer risks and screening practices among women living in a resource poor community in Tshwane, South Africa. Breast J 19:453-454, 2013 23721568 10.1111/tbj.12143

[8] De Ver Dye T Bogale S Hobden C et al: A mixed-method assessment of beliefs and practice around breast cancer in Ethiopia: Implications for public health programming and cancer control. Glob Public Health 6:719-731, 2011 20865612 10.1080/17441692.2010.510479

[9] Clegg-Lamptey J Dakubo J Attobra YN: Why do breast cancer patients report late or abscond during treatment in Ghana? A pilot study. Ghana Med J 43:127-131, 2009 20126325 PMC2810246

[10] Ezeome ER: Delays in presentation and treatment of breast cancer in Enugu, Nigeria. Niger J Clin Pract 13:311-316, 2010 20857792

[11] O’Brien KS Soliman AS Annan K et al: Traditional herbalists and cancer management in Kumasi, Ghana. J Cancer Educ 27:573-579, 2012 22549472 10.1007/s13187-012-0370-z PMC4276030

[12] Odigie VI Tanaka R Yusufu LM et al: Psychosocial effects of mastectomy on married African women in northwestern Nigeria. Psychooncology 19:893-897, 2010 20025083 10.1002/pon.1675

[13] Parham GP Mwanahamuntu MH Kapambwe S et al: Population-level scale-up of cervical cancer prevention services in a low-resource setting: Development, implementation, and evaluation of the cervical cancer prevention program in Zambia. PLoS One 10:e0122169, 2015 25885821 10.1371/journal.pone.0122169 PMC4401717

[14] Warrier S Tapia G Goltsman D et al: An update in breast cancer screening and management. Womens Health (Lond) 12:229-239, 2016 26689336 10.2217/whe.15.105 PMC5375048

[15] US Preventive Services Task Force: Screening for breast cancer: US Preventive Services Task Force recommendation statement. Ann Intern Med 151:716-726, W-236, 2009 10.7326/0003-4819-151-10-200911170-00008 19920272

[16] Berg WA Zhang Z Lehrer D et al: Detection of breast cancer with addition of annual screening ultrasound or a single screening MRI to mammography in women with elevated breast cancer risk. JAMA 307:1394-1404, 2012 22474203 10.1001/jama.2012.388 PMC3891886

[17] Miller AB: Early detection of breast cancer in the emerging world. Zentralbl Gynakol 128:191-195, 2006 16835812 10.1055/s-2006-933488

[18] Miller AB To T Baines CJ et al: Canadian National Breast Screening Study-2: 13-year results of a randomized trial in women aged 50-59 years. J Natl Cancer Inst 92:1490-1499, 2000 10995804 10.1093/jnci/92.18.1490

[19] Miller AB To T Baines CJ et al: The Canadian National Breast Screening Study-1: Breast cancer mortality after 11 to 16 years of follow-up—A randomized screening trial of mammography in women age 40 to 49 years. Ann Intern Med 137:305-312, 2002 12204013 10.7326/0003-4819-137-5_part_1-200209030-00005

[20] Sankaranarayanan R Ramadas K Thara S et al: Clinical breast examination: Preliminary results from a cluster randomized controlled trial in India. J Natl Cancer Inst 103:1476-1480, 2011 21862730 10.1093/jnci/djr304

[21] Anderson BO Ilbawi AM El Saghir NS: Breast cancer in low and middle income countries (LMICs): A shifting tide in global health. Breast J 21:111-118, 2015 25444441 10.1111/tbj.12357

[22] Carlson RW Allred DC Anderson BO et al: Invasive breast cancer. J Natl Compr Canc Netw 9:136-222, 2011 21310842 10.6004/jnccn.2011.0016

[23] Yang W Dempsey PJ: Diagnostic breast ultrasound: Current status and future directions. Radiol Clin North Am 45:845-861, vii, 2007 17888773 10.1016/j.rcl.2007.06.009

[24] Vargas HI Anderson BO Chopra R et al: Diagnosis of breast cancer in countries with limited resources. Breast J 9:S60-S66, 2003 12713498 10.1046/j.1524-4741.9.s2.5.x

[25] Raza S Goldkamp AL Chikarmane SA et al: US of breast masses categorized as BI-RADS 3, 4, and 5: Pictorial review of factors influencing clinical management. Radiographics 30:1199-1213, 2010 20833845 10.1148/rg.305095144

[26] Shyyan R Sener SF Anderson BO et al: Guideline implementation for breast healthcare in low- and middle-income countries: Diagnosis resource allocation. Cancer 113:2257-2268, 2008 18837018 10.1002/cncr.23840

[27] White RR Halperin TJ Olson JA Jr et al: Impact of core-needle breast biopsy on the surgical management of mammographic abnormalities. Ann Surg 233:769-777, 2001 11371735 10.1097/00000658-200106000-00006 PMC1421319

[28] Dalberg K Eriksson E Kanter L et al: Biomarkers for local recurrence after breast-conservation: A nested case-control study. Breast Cancer Res Treat 57:245-259, 1999 10617301 10.1023/a:1006281718793

[29] Newman LA Kuerer HM: Advances in breast conservation therapy. J Clin Oncol 23:1685-1697, 2005 15755977 10.1200/JCO.2005.09.046

[30] Ntirenganya F Petroze RT Kamara TB et al: Prevalence of breast masses and barriers to care: Results from a population-based survey in Rwanda and Sierra Leone. J Surg Oncol 110:903-906, 2014 25088235 10.1002/jso.23726

[31] Yip CH Buccimazza I Hartman M et al: Improving outcomes in breast cancer for low- and middle-income countries. World J Surg 39:686-692, 2015 25398564 10.1007/s00268-014-2859-6

[32] Chatterjee A Serniak N Czerniecki BJ: Sentinel lymph node biopsy in breast cancer: A work in progress. Cancer J 21:7-10, 2015 25611773 10.1097/PPO.0000000000000090 PMC4304410

